# Ribonuclease 1 Enhances Antitumor Immunity against Breast Cancer by Boosting T cell Activation

**DOI:** 10.7150/ijbs.84592

**Published:** 2023-06-04

**Authors:** Ying-Nai Wang, Heng-Huan Lee, Zhou Jiang, Li-Chuan Chan, Gabriel N. Hortobagyi, Dihua Yu, Mien-Chie Hung

**Affiliations:** 1 Department of Molecular and Cellular Oncology, The University of Texas MD Anderson Cancer Center, Houston, TX 77030, USA.; 2 Department of Lymphoma and Myeloma, The University of Texas MD Anderson Cancer Center, Houston, TX 77030, USA.; 3 Department of Breast Medical Oncology, The University of Texas MD Anderson Cancer Center, Houston, TX 77030, USA.; 4 UTHealth Graduate School of Biomedical Sciences, The University of Texas MD Anderson Cancer Center, Houston, TX 77030, USA.; 5 Graduate Institute of Biomedical Sciences, Institute of Biochemistry and Molecular Biology, Research Center for Cancer Biology, Cancer Biology and Precision Therapeutics Center, and Center for Molecular Medicine, China Medical University, Taichung 406, Taiwan.; 6 Department of Biotechnology, Asia University, Taichung, 413, Taiwan.

**Keywords:** breast cancer, ribonuclease 1, tumor microenvironment, T cell activity, antitumor immunity, tumor-infiltrating immune cells

## Abstract

The secretory enzyme human ribonuclease 1 (RNase1) is involved in innate immunity and anti-inflammation, achieving host defense and anti-cancer effects; however, whether RNase1 contributes to adaptive immune response in the tumor microenvironment (TME) remains unclear. Here, we established a syngeneic immunocompetent mouse model in breast cancer and demonstrated that ectopic RNase1 expression significantly inhibited tumor progression. Overall changes in immunological profiles in the mouse tumors were analyzed by mass cytometry and showed that the RNase1-expressing tumor cells significantly induced CD4^+^ Th1 and Th17 cells and natural killer cells and reduced granulocytic myeloid-derived suppressor cells, supporting that RNase1 favors an antitumor TME. Specifically, RNase1 increased expression of T cell activation marker CD69 in a CD4^+^ T cell subset. Notably, analysis of cancer-killing potential revealed that T cell-mediated antitumor immunity was enhanced by RNase1, which further collaborated with an EGFR-CD3 bispecific antibody to protect against breast cancer cells across molecular subtypes. Our results uncover a tumor-suppressive role of RNase1 through adaptive immune response in breast cancer *in vivo* and *in vitro*, providing a potential treatment strategy of combining RNase1 with cancer immunotherapies for immunocompetent patients.

## Introduction

Breast cancer is the most diagnosed cancer worldwide in females and the leading cause of cancer-related death in the United States among women aged 20 to 59 years 1. The use of adjuvant therapies after surgery, including radiotherapy, chemotherapy, targeted therapy, hormone therapy, and immunotherapy, has led to lower breast cancer mortality rates 2. However, a significant number of patients with breast cancer, in particular those with basal-like breast cancer, which lacks expression of estrogen and progesterone receptors and human epidermal growth factor receptor 2 (HER2), still face a poor prognosis with a high risk of recurrence or drug resistance 3, 4. Hence, optimal strategies for enhancing therapeutic efficacy against breast tumors are urgently needed 5. It is now understood that the host immune system in the tumor microenvironment (TME) influences breast cancer outcomes 6, 7. High levels of tumor-infiltrating lymphocytes that comprise CD4^+^ helper and CD8^+^ cytotoxic T cells correlate with a better prognosis in patients with basal-like and HER2-positive breast cancer 8-10, suggesting that immune activation in modulating the TME favors host antitumor immunity, which points to a promising modality for fighting breast cancer.

Ribonuclease (RNase) 1 belongs to the family of pancreatic-type endoribonucleases called the human RNase A superfamily, which includes 13 members closely located on chromosome 14 and divided into two subgroups, canonical RNases 1-8 and noncanonical RNases 9-13 11, 12. All the RNase genes encode secretory proteins containing an N-terminal hydrophobic signal peptide; among them, RNase1 is the most broadly expressed in various tissues and secreted abundantly in the circulatory system such as serum and plasma 13, 14. RNase1 is recognized as a catabolic enzyme for its ribonucleolytic activity to degrade single- and double-stranded RNAs and RNA:DNA hybrids 15, 16. RNase1 is known to cleave viral RNAs and exert antiviral activity, which contributes to the innate immune response against viral pathogens 17. Of note, secretory RNase1 has a predominant role in the clearance of RNAs in the extracellular space. Studies of RNase1 as a scavenger of extracellular RNAs (exRNAs) have indicated its vessel protective role to be anti-coagulation in ischemic cardiovascular diseases, including myocardial infarction and atherosclerosis 18-20. Furthermore, RNase1 as an anti-inflammatory mediator cleaves excess exRNAs to prevent exRNAs-induced elevation of proinflammatory cytokines, such as tumor necrosis factor-α, in cardiovascular pathophysiology and in a xenograft mouse model of colon carcinoma HT-29 cells, leading to cardiomyocyte function protection and tumor regression, respectively 21-23.

It has recently been determined that RNase1 serves as a ligand of the receptor tyrosine kinase ephrin receptor A4 (EphA4), which is independent of its ribonucleolytic activity and exRNAs are not involved in this role 24. Similarly, two other RNase proteins, RNase5 and RNase7, have cognate receptors of EGFR in pancreatic cancer and ROS1 in liver cancer, respectively, and neither of these roles involves ribonucleolytic activity 25, 26. Studies in breast cancer showed that secretory RNase1 binds to and activates EphA4 signaling and the RNase1-EphA4-activating axis promotes stem cell-like properties and tumor growth in nude mouse models 24. Structural analysis revealed that the binding site of RNase1 on EphA4 partially overlaps with the site on EphA4 that binds to ephrin-A5, a classical ligand of EphA4, due to steric hindrance 27. Notably, EphA4 is expressed not only on tumor cells but also on immune cells in the TME, including T and B lymphocytes 28-30.

With the understanding that RNase1 is critical for innate immunity, in this study, we reveal an important role of RNase1 in the adaptive immune system, namely that RNase1 increases the frequencies of specific subsets of tumor-infiltrating immune cells, including T cells, and boosts T cell activation, leading to an antitumor TME in breast cancer.

## Materials and Methods

**Cell culture and treatment.** The breast cancer cell lines BT-549, MDA-MB-231, MCF7, SKBR3, MDA-MB-453, 293T, Jurkat, and 4T1 were obtained from American Type Culture Collection. All cell lines tested mycoplasma negative, validated by short-term repeat DNA fingerprinting at The University of Texas MD Anderson Cancer Center and were maintained in Dulbecco's modified Eagle's medium/F12 supplemented with 10% fetal bovine serum. Human PBMC (#70025) and primary T cells (#70024) were purchased from STEMCELL Technologies and cultured in RPMI 1640 medium containing 10% fetal bovine serum and IL-2 (10 ng/ml; Miltenyi Biotec, #130-097-744) to maintain T cell activity. Treatment with recombinant RNase1 protein purified from HEK293 cells (Sino Biological Inc. #13468-H08H-100) in the *in vitro* experiments was carried out at a concentration of 1 μg/ml. The concentrations used of EGFR-CD3 bsAb (G&P Biosciences, #FCL2954) and recombinant His-A4 protein (Sino Biological, #11314-H08H) were 1 μg/ml and 5 μg/ml, respectively.

**Cell lysis and Western blotting.** Cells were washed with phosphate-buffered saline (PBS) twice and then lysed in RIPA lysis buffer containing 150 mM NaCl, 50 mM Tris (pH 7.5), 1% Nonidet P-40, phosphatase inhibitor cocktail (Bimake, #B15002), and protease inhibitor cocktail (Bimake, #B14002). After sonication and centrifugation at 14,000*g* for 10 min, the supernatants were collected and protein concentrations were determined by Thermo Scientific Pierce BCA Protein Assay (Thermo Fisher Scientific, #PI-23227). Protein samples were loaded and separated by SDS-PAGE gel electrophoresis, transferred onto a PVDF membrane, and subjected to Western blotting using the indicated antibodies at 4 °C incubation overnight. Data detection was performed using chemiluminescent detection reagents (Bio-Rad #170-5061) and ImageQuant LAS 4010 (GE Healthcare) according to the manufacturer's instructions. Primary antibodies used in this study were RNase1 (1:1,000; Sigma-Aldrich, #HPA001140), CD14 (1:1,000; Cell Signaling Technology, #56082), and tubulin (1:10,000; Sigma-Aldrich, #T5168).

**Generation of membrane-bound anti-CD3 antibody fragment-expressing stable cells.** The nucleotide sequence encoding membrane-bound anti-CD3 antibody fragment (GenBank: accession HM208750.1, CD5L-OKT3scFv-CD14 protein gene) 31 was synthesized from Genewiz/Azenta Life Sciences, and the expression vector was generated by insertion of the fragment into the lentiviral vector pCDH-CMV-MCS-EF1-NEO (System BioSciences, #CD514B-1). To establish BT-549 stable cell lines expressing membrane-bound anti-CD3 antibody fragment (BT-549-αCD3) or the control counterpart of neomycin/G418 selection vector (BT-549-Vn), we conducted lentiviral packaging via transient transfection of 1 μg of pCDH-αCD3 or pCDH-NEO empty vector with 1 μg of pCMV-VSVG and 0.5 μg of pCMV-dR8.91 expression plasmids in 5 × 10^5^ 293T cells. After 72 h, conditioned medium from the 293T transfectants was collected, centrifuged at 6,000*g* for 15 min, and flew through a 0.45-μm filter, followed by incubating BT-549 cells at 5 μg/ml polybrene (EMD Millipore, #TR-1003-G) for lentiviral transduction. After a 16-h transduction, cells were replenished with complete medium for 24 h and subjected to G418 selection (400 μg/ml; Thermo Fisher Scientific, #11811031) to establish stable cells. To generate stable clones exogenously expressing RNase1 or the control counterpart of puromycin selection vector in BT-549-Vn or BT-549-αCD3 cells, we conducted lentiviral packaging via transient transfection of 1 μg of pCDH-R1 24 or pCDH-puro empty vector with 1 μg of pCMV-VSVG and 0.5 μg of pCMV-dR8.91 expression plasmids in 5 × 10^5^ 293T cells and performed lentiviral transduction in BT-549-Vn or BT-549-αCD3 cells. The stable clones were established by puromycin selection (1 μg/ml; InvivoGen Corporation, #ant-pr-1). All stable transfectants were selected from a pool of clones. The duration of stable cell selection was at least 3 weeks.

**Animal studies.** All animal experiments were performed under the guidelines and the institutional animal care protocol (00001250-RN02) approved by the Institutional Animal Care and Use Committee at MD Anderson Cancer Center. Mice were maintained at humidity levels between 30% and 70% and temperature range of 70 ± 2 °F (21.1 ± 1.1 °C) under a 12-h light/12-h dark cycle. No statistical method of the experiment was used to estimate sample size. Six-week-old female BALB/c and J:NU mice were purchased from The Jackson Laboratory. The mouse 4T1-vector or 4T1-RNase1 stable clones (5 × 10^4^) were suspended in 50 μl of Dulbecco's modified Eagle's medium/F12 (Corning, #10-090-CV) mixed with 50 μl of Matrigel (Thermo Fisher Scientific, #CB40230C). The cell mixtures were then orthotopically injected into the mammary fat pads of mice. Compound 1 (50 mg/kg dissolved in 5% DMSO with 10% Tween 80 in PBS; Santa Cruz Biotechnology, #sc-314230) or a matched vehicle was given on day 7 (for BALB/c) or day 10 (for J:NU) after cell injection by intraperitoneal administration for a total of ten (for BALB/c) or eight (for J:NU) treatments. Tumor volume was determined *in vivo* by external caliper and calculated using the formula volume = (length × width^2^)/2. Tumor weight was measured at the endpoint.

**CyTOF.** Tumors were harvested from mice and digested to generate single-cell suspensions using Tumor Dissociation Kit (Miltenyi Biotec, #130-096-730) and gentleMACS Dissociator (Miltenyi Biotec) following the manufacturer's instructions. Prior to immunostaining with CyTOF antibodies, which were prepared by the Flow Cytometry and Cellular Imaging Core Facility at MD Anderson, the cells were incubated with TruStain FcX anti-mouse CD16/CD32 antibody (BioLegend, #101319) for 15 min for blocking nonspecific binding of IgG Fc receptors. The cells were then stained with cocktail mixes of metal-conjugated antibodies to cell-surface proteins. After washing, the cells were fixed and permeabilized with methanol and stained with cocktail mixes of metal-conjugated antibodies to intracellular proteins. The samples were washed in PBS and resuspended in double-distilled water containing bead standards, followed by mass cytometric analysis (Fluidigm). The acquired data were normalized and manually gated using FlowJo software (version 10.8.1) to exclude beads, dead cells, debris, and doublets. To identify immune profiles, a total of 180,000 events across all 18 samples with equal event sampling using 10,000 events per sample were then subjected to t-SNE 32 and FlowSOM 33 clustering analysis in FlowJo. A heatmap of marker intensities was generated in R package according to the mean value for each marker in all clusters. Statistical analysis and graphing of population frequencies were performed with GraphPad Prism software (version 9).

**Flow cytometric analysis.** To analyze the effect of RNase1 on T cell activation in the TME, adherent BT-549 stable tumor cells were seeded and cultured overnight. The pre-seeded BT-549 cells were then cocultured with Jurkat cells or PBMCs at a 1:5 ratio (tumor cell:Jurkat or PBMCs) for 24 h, in the presence or absence of RNase1 treatment. Jurkat cells or PBMCs were collected and stained with fluorescence-labeled antibodies of interest in 100 μl of staining buffer (BioLegend, #420201) at 4 °C for 30 min in the dark. The cells were then washed with the staining buffer twice, resuspended in 500 μl of staining buffer, and subjected to flow cytometry. Antibodies used for surface protein staining include PE-Cy7-conjugated anti-CD69 (1:100; BioLegend, #310912), APC-conjugated anti-CD3 (1:50; BioLegend, #300312), PE-conjugated anti-CD4 (1:100; BioLegend, #317410), and FITC-conjugated anti-CD8 (1:100; BioLegend, #301006), with the use of PE-Cy7 mouse IgG1 (1:200; BioLegend, #400125), APC mouse IgG2a (1:200; BioLegend, #400219), PE mouse IgG2b (1:200; BioLegend, #400313), and FITC mouse IgG1 (1:200; BioLegend, #400104) as control staining, respectively. For analyzing EphA4 expression on PBMC-T cells, 2 × 10^6^ PBMCs were stained with APC-conjugated anti-CD3 or IgG isotype control in 100 μl of staining buffer at 4 °C for 30 min in the dark. After washing with PBS twice, the cells were fixed by fixation buffer (R&D Systems, #FC009) for 10 min at room temperature in the dark, washed twice with PBS, and resuspended the cell pellet in 100 μl of permeabilization/wash buffer (R&D Systems, #FC009) with or without anti-EphA4 antibody (1:10; Proteintech, #21875-1-AP) at 4 °C for 30 min in the dark. The cells were then washed by permeabilization/wash buffer and stained with anti-rabbit secondary antibody at 4 °C for 20 min in the dark. After washing with staining buffer twice, the cells were resuspended in 500 μl of staining buffer and subjected to flow cytometry. Stained cells were analyzed with data acquired by a BD FACSCanto II flow cytometer using BD FACSDiva v8.0.2 software and processed in FlowJo v10.8.1 software (BD Biosciences).

**T cell-mediated tumor cell-killing assay.** BT-549 stable tumor cells (5 × 10^3^ cells in 100 μl of medium) were seeded in a 96-well plate and cultured overnight. The tumor cells were then cocultured with PBMCs or primary T cells at a 1:10 ratio containing caspase 3/7 substrate green dye (1:1000; Sartorius, #4440), in the presence or absence of RNase1 treatment. Green fluorescent signals and phase object confluence were measured and images were captured every 2 h in the Incucyte live-cell analysis system (Essen Bioscience). Data were analyzed using the integrated software.

**Analysis of EphA4 expression from databases.** An open-access database, Human Protein Atlas, was used to analyze the gene expression of EphA4. Images and data were available from v22.proteinatlas.org, including a variety of immune cells based on RNA-sequencing results from Monaco 34 and Schmiedel 35 datasets and PBMCs analyzed on a single-cell level or using flow-sorted data.

**Statistical analysis.** Data are presented as mean ± standard deviation (SD) or standard error of mean (SEM) as stated in Figure legends. An unpaired *t*-test or ANOVA analysis was used to compare the continuous variables between groups. A p value of < 0.05 was considered statistically significant. Mass and flow cytometry results were analyzed using FlowJo Software (version 10.8.1; BD Life Sciences). Statistical analyses were graphed using GraphPad Prism program (version 9; GraphPad Software Inc.).

## Results

### RNase1 ectopic expression inhibits tumor progression in an immunocompetent mouse model

**Figure 1:**
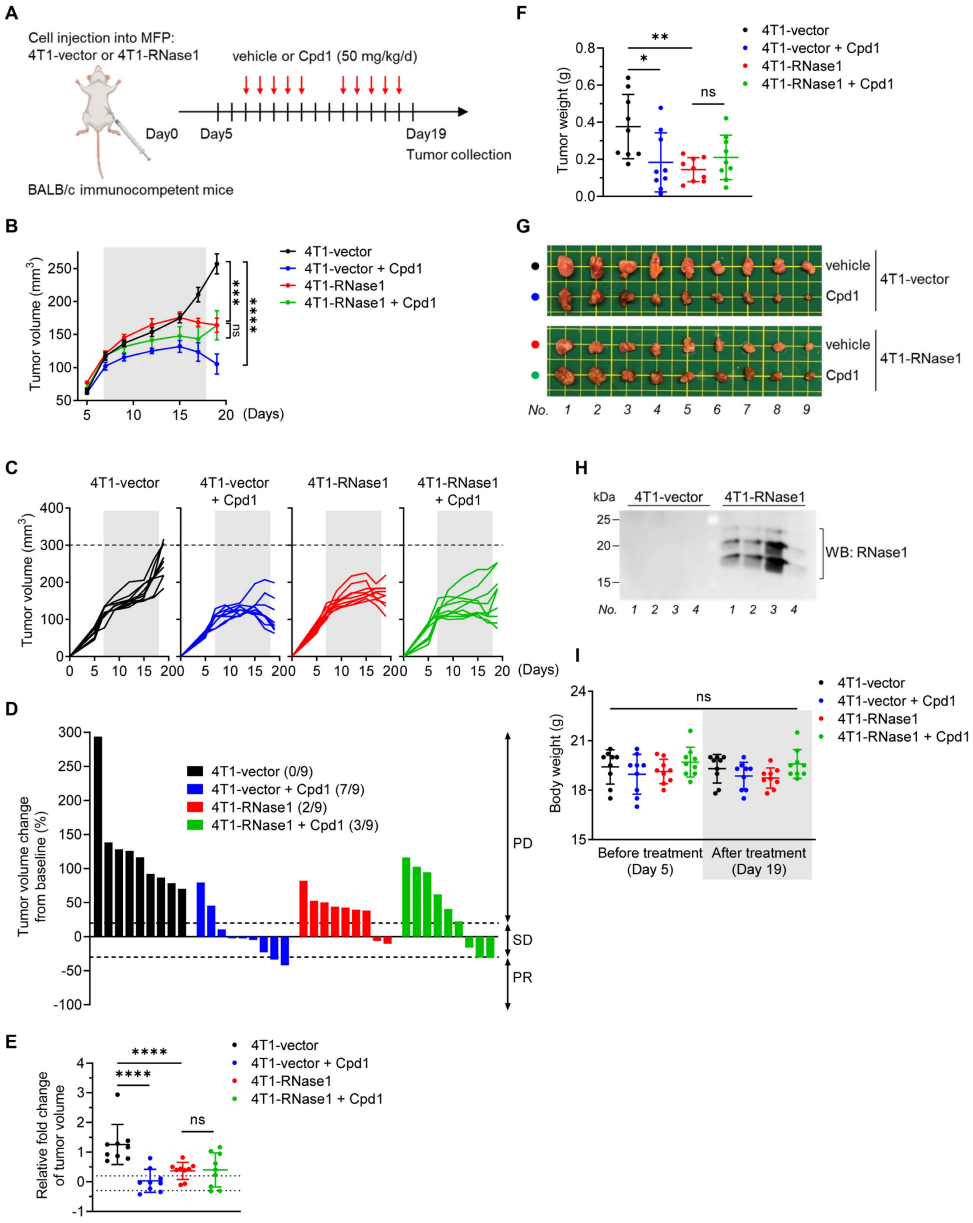
** RNase1 ectopic expression inhibits tumor progression in an immunocompetent mouse model.** (A) The schedule of treatment in BALB/c mice orthotopically injected with the indicated 4T1 cells into the mammary fat pad (MFP) followed by treatment with vehicle or compound 1 (Cpd1). The arrows indicate the time of treatment. n = 9 mice per group. (B-D) Average tumor volume (B), tumor growth curve (C), and waterfall plot analysis (D) of 4T1 cells in BALB/c mice treated with vehicle or Cpd1 as indicated. Tumor volume was measured at the indicated time points, and tumors were dissected at the end point. The gray box indicates the duration of treatment. The waterfall plot generated from the tumor growth curve indicates tumor progression in each mouse under individual treatment. The number of mice that experienced tumor progression in each group is shown in parentheses. (E-G) Relative fold changes of tumor volume (E), tumor weight (F), and representative images (G) from (C). (H) Western blotting of the indicated mice tumors with antibodies against RNase1. The experiment was repeated a second time with similar results. (I) Measurement of body weight before and after treatment as indicated. The gray box indicates the body weight at the end point (i.e., day 19). Data represent mean ± SD (E, F, and I) or mean ± SEM (B). *p < 0.05, **p < 0.01, ***p < 0.001, ****p < 0.0001, ns, not significant, ANOVA test. PD, progressive disease; SD, stable disease; PR, partial response.

To determine the role of RNase1 in the TME of breast cancer, we utilized a set of established stable clones 24, namely mouse 4T1 mammary tumor cells expressing mouse RNase1 (4T1-RNase1) and vector control (4T1-vector), and orthotopically implanted the cells into the mammary fat pad of immunocompetent BALB/c mice (Figure 1A). Significantly, in the presence of the immune system, the 4T1 tumor-bearing mice with RNase1 ectopic expression exhibited less tumor volume than did the control mice (Figure 1B and 1C; red vs. black). We graphed the tumor volume as a continuous variable in a waterfall plot, which showed that RNase1-expressing 4T1 tumor-bearing mice had a higher response rate compared to the control mice (Figure 1D). Similar results were observed in the relative fold changes of tumor volume (Figure 1E) and tumor weight (Figure 1F; representative images, Figure 1G). A Western blot analysis confirmed the expression of RNase1 in the 4T1-RNase1 group (Figure 1H), whose overall health appeared good during the course of treatment, as their body weight did not significantly differ from that of the 4T1-vector group (Figure 1I). Unlike previous findings showing that RNase1 increases tumor weight in J:NU immunocompromised nude mice 24 who lack functional T lymphocytes but preserve lineages such as macrophages and natural killer (NK) cells, our results surprisingly suggest that RNase1-mediated tumor shrinkage occurs in a lymphocyte-dependent manner in the syngeneic tumor system.

As shown in Figure S1A to S1C, consistent with nude mice's tumor weight data 24, we detected a significant induction of tumor volume in nude mice bearing 4T1-RNase1 tumors compared with the mice injected with control tumor cells (red vs. black); the induction was suppressed by the EphA4 inhibitor compound 1, which binds to the ligand-binding domain of EphA4 and EphA2 and inhibits their receptor activation 36 (red vs. green).

These results support the previous findings that the RNase1-EphA4-activating axis contributes to breast tumor progression in an immunocompromised mouse model, in which there was no significant difference between 4T1-vector mice and 4T1-vector plus compound 1 mice in tumor volume (Figure S1B, black vs. blue) or weight 24. However, interestingly, in immunocompetent BALB/c mice injected with the same 4T1-stable clones, the 4T1-vector mice treated with compound 1 had significantly repressed tumor volume and tumor weight and an enhanced response rate, compared with those not treated with compound 1 (Figure 1B to 1G, blue vs. black). These results from two different model systems strongly suggest that the ability (e.g. receptor clustering and/or signaling complex formation) of EphA4 forward signals into tumor cell mediated by RNase1 or compound 1 is distinct form the signals into immune cells. Furthermore, in the 4T1-RNase1 BALB/c mice, the addition of compound 1 had no synergistic impact of RNase1 in the antitumor effect (Figure 1B to 1G, red vs. green), suggesting that RNase1 and compound 1 may target a common profile to contribute to the tumor-suppressive function, probably owing to conformational changes upon binding to EphA4 and transmission of EphA4 forward signals into immune cells to unleash antitumor immunity.

### Expression of RNase1 in mice favors an antitumor immunological profile in the TME

**Figure 2:**
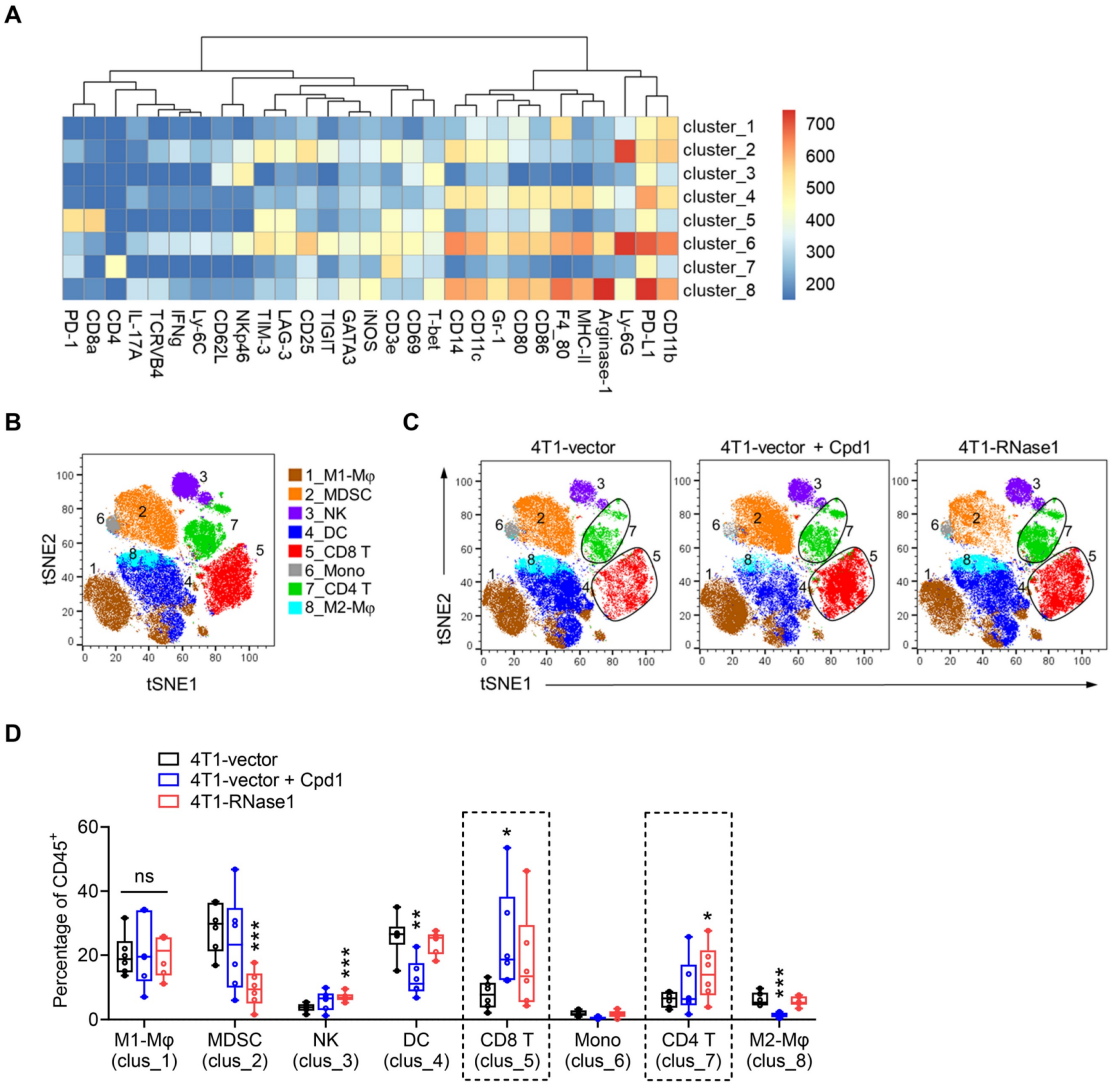
** Mice expressing RNase1 favor an antitumor TME.** (A) A heatmap of different marker intensities in CD45^+^ tumor-infiltrating immune cell clusters identified by CyTOF data analysis using t-SNE and FlowSOM. (B and C) Annotation of t-SNE plots of immune cells overlaid with color-coded clusters based on marker expression as shown in (A). Mφ, macrophage; MDSC, myeloid-derived suppressor cell; NK, natural killer cell; DC, dendritic cell; CD8 T, CD8^+^ T cell; Mono, monocytes; CD4 T, CD4^+^ T cell. (D) Box plots of frequency of CD45^+^ tumor-infiltrating immune cell clusters as indicated. n = 6 mice per group. Box plots indicate minima (lower end of whisker), maxima (upper end of whisker), median (center), 25th percentile (bottom of box), and 75th percentile (top of box). *p < 0.05, **p < 0.01, ***p < 0.001, individually compared with the percentage at the first bar of each cluster (4T1-vector), one-tailed unpaired *t* test.

Next, we examined the overall changes in the TME immunological profile that may participate in the antitumor effect mediated by compound 1 or RNase1. To this end, we analyzed tumor-infiltrating cells among 4T1-vector, 4T1-vector plus compound 1, and 4T1-RNase1 tumors using mass cytometry by time-of-flight (CyTOF) 37, 38 with a panel of antibodies against various immune cell markers, followed by t-distributed stochastic neighbor embedding (t-SNE) 32 and FlowSOM 33 clustering analysis (Figure 2). A clustering algorithm of CD45^+^ immune cells from tumors identified eight major immune cell clusters; we then manually annotated these clusters based on marker expression (Figure 2A and 2B). Visually, clusters 5 and 7, representing CD8^+^ T cells and CD4^+^ T cells, respectively, exhibited increases in the compound 1 and RNase1 groups compared with the vector control group without compound 1 (Figure 2C and S2).

As shown in Figure 2D, a quantitative analysis based on the t-SNE-guided population indicated a remarkable increase in CD8^+^ T cell frequency in tumors from mice treated with compound 1 (clus_5, blue vs. black); CD8^+^ T cells tended to increase in samples from 4T1-RNase1 mice in a trend toward statistical significance (clus_5, red vs. black). Notably, the CD4^+^ T cell frequency in 4T1-RNase1 tumors was significantly increased compared to that of 4T1-vector control (Figure 2D, clus_7, red vs. black). The expansions of the T cell populations suggest a critical role of compound 1-mediated or RNase1-mediated antitumor response. Moreover, tumors harboring RNase1 ectopic expression had decreased frequencies of myeloid-derived suppressor cells (MDSCs) known to inhibit T cell activity 39 and an increase in NK cells 40 (Figure 2D, clus_2 and _3, respectively, red vs. black), implicating T cell responses regulated by RNase1. Together with the results that 4T1-RNase1 tumor samples did not have changes in the frequencies of M1-like and M2-like macrophages, dendritic cells (DCs), and monocytes (Figure 2D, clus_1, _8, _4, and _6, respectively, red vs. black), cells expressing RNase1 favor an antitumor TME, possibly due to the interplay between T and NK cells as well as MDSCs.

In response to compound 1 treatment, in addition to the above-mentioned CD8^+^ T cell expansion, we observed an increased frequency of NK cells in a near-significant trend (Figure 2D, clus_3, blue vs. black) and a decreased frequency of pro-tumorigenic M2-like macrophages 41 (clus_8, blue vs. black), which supports tumor suppression due to compound 1 treatment. However, we also noted a decreased level of DCs upon compound 1 treatment (clus_4, blue vs. black), and DCs are thought to support antitumor activity 42. The mechanism of such a reduction of DCs remains unknown. Because compound 1, in addition to its EphA4 inhibition, also interacts with the ligand-binding domain of EphA2 36, we speculate that this discrepancy might result from an undefined mechanism related to the compound 1-affected dysfunction of EphA2, whose expression is harbored mainly on the DCs of the immune system 28, 43.

EphA4 is the common target previously reported to interact with both RNase1 24 and compound 1 36, and its expression is not only on tumor cells but also on a variety of immune cells in the TME 28. To minimize the issues that might be caused by the compound 1-EphA2 association, hereinafter we focused on RNase1 to further pursue how RNase1 contributes to antitumor immunity and whether EphA4 is involved in RNase1-mediated tumor suppression in the breast TME.

### RNase1 targets specific subsets of tumor-infiltrating immune cells

**Figure 3:**
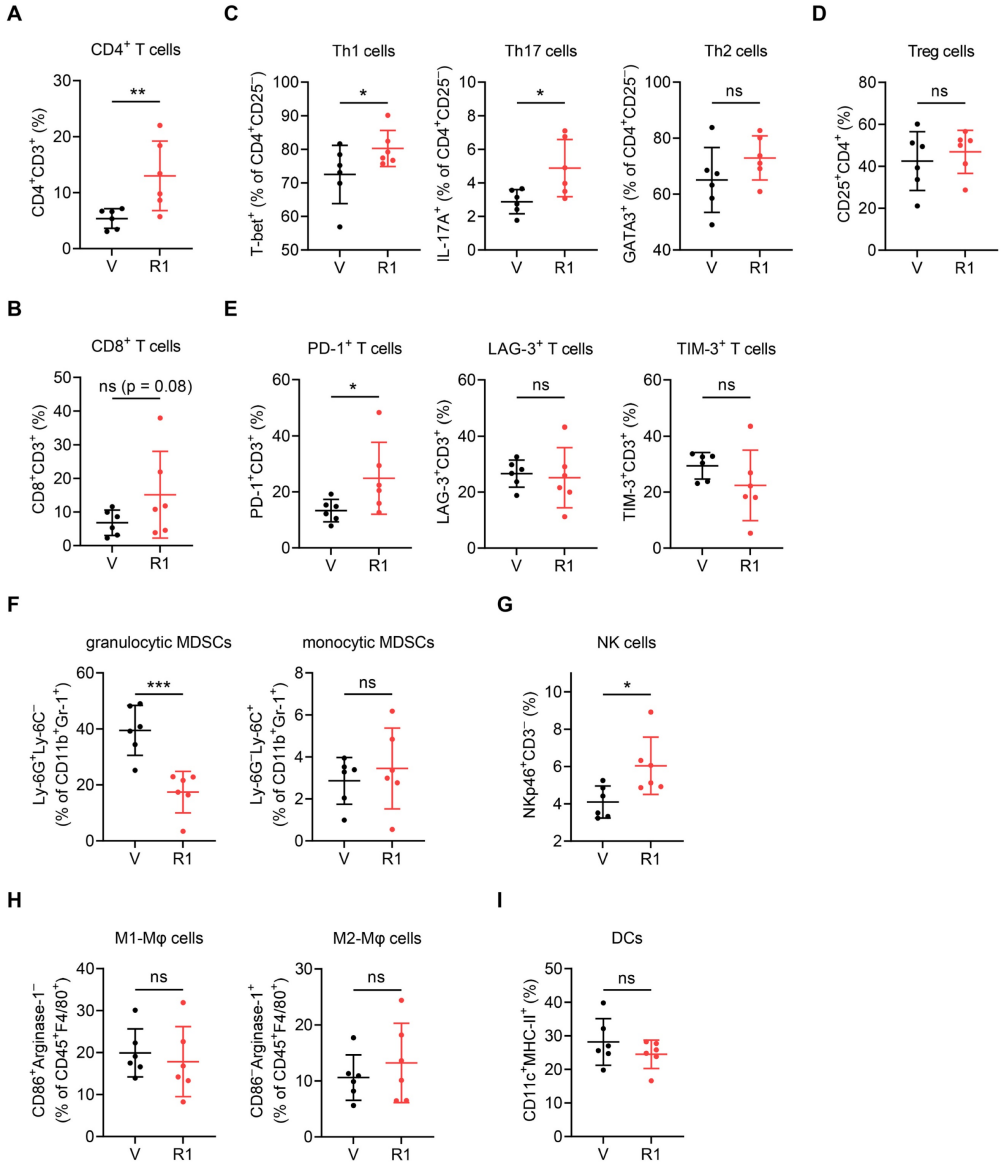
** RNase1 targets specific subsets of tumor-infiltrating immune cells using a conventional hand-gated strategy.** Frequency of the indicated subsets in total CD45^+^ tumor-infiltrating immune cells, including CD4^+^ T cells (A), CD8^+^ T cells (B), T helper cells (C), Treg cells (D), immune checkpoint receptors (E), granulocytic and monocytic MDSCs (F), NK cells (G), M1- and M2-like macrophages (H), and DCs (I). Data represent the mean ± SD. n = 6 mice per group. *p < 0.05, **p < 0.01, ***p < 0.001, ns, not significant, one-tailed unpaired *t* test.

Consistent with the above data based on the t-SNE-guided stratification (Figure 2), a conventional hand-gated strategy (Figure S3) showed that RNase1 increased the frequency of the CD4^+^ T cell subset by greater than two-fold and the CD8^+^ T cell subset with a trend toward significance (Figure 3A and 3B). Among the CD4^+^CD25^-^ T helper (Th) cell population, we found that RNase1 expanded the frequencies of Th1 (T-bet^+^) and Th17 (IL-17A^+^) cells, whereas the frequency of Th2 (GATA3^+^) cells was not obviously altered (Figure 3C). Moreover, there was no significant difference in the levels of immunosuppressive Treg cells 44 (CD4^+^CD25^+^, Figure 3D). Th1 and Th2 cells operate as antitumor and protumor effector cells, respectively 45; and although Th17 cells have both antitumorigenic and pro-tumorigenic roles, Th17 cells can exert antitumor activities by increasing NK and T cell infiltration into the TME 46, 47. Hence, our results indicate that the increased cell frequencies of Th1 and Th17, independent of Th2 and Treg cells, dominate T cell-mediated antitumor immunity promoted by RNase1, strongly supporting our notion that the existence of RNase1 enhances antitumor immune system to inhibit breast tumorigenesis. Furthermore, we detected increased levels of programmed cell death 1 (PD-1) on T cells in response to RNase1 but no changes in lymphocyte activation gene 3 (LAG-3) or T cell immunoglobulin mucin-3 (TIM-3) (Figure 3E). Researchers have demonstrated that PD-1 expression in itself can be considered a marker of activated tumor-reactive T cells 48, while exhausted T cells that are highly associated with CD8^+^ T cell dysfunction gain co-expression of multiple checkpoint inhibitory receptors including PD-1, TIM-3, and LAG-3 49, 50. As the sole PD-1 upregulation shown in Figure 3E, cells expressing RNase1 in the TME preferentially drive T cell activation.

We examined the cell frequency of immunosuppressive MDSCs identified within the CD11b^+^Gr-1^+^ population and found a striking RNase1-mediated decrease in granulocytic MDSCs, which represent more than 80% of all MDSCs in most types of cancer and primarily cause antigen-specific T cell suppression, but we found no change in monocytic MDSCs, which suppress T cell responses in both antigen-specific and antigen-nonspecific manners not requiring cellular contact 51, 52 (Figure 3F, Ly-6G^+^Ly-6C^-^ vs. Ly-6G^-^Ly-6C^+^). In addition, the presence of RNase1 led to an increased population of NK cells (Figure 3G, NKp46^+^CD3^-^), and no significant alterations were observed in the frequencies of M1-like (F4/80^+^CD86^+^Arginase-1^-^) or M2-like (F4/80^+^CD86^-^Arginase-1^+^) macrophages and DCs (CD11c^+^MHC-II^+^) (Figure 3H and 3I). These results match those gated by t-SNE (Figure 2D). Collectively, these results showed that RNase1 contributed to a significant induction of CD4^+^ Th1, CD4^+^ Th17, and NK cells, which associate with antitumor immunity, as well as a reduction of immunosuppressive MDSCs that associate with pro-tumor activity. It is known that CD4^+^ T cells are critical for the initiation of NK cell activation in lymph nodes 53 and that T cells are the main targets of MDSCs implicated in immunosuppression 51. Considering that both the BALB/c and the J:NU mouse models used in this study harbor NK cell lineages, we next aimed to determine whether T cells act as major mediators in RNase1-regulated tumor suppression.

### RNase1 boosts CD4^+^ T cell activation

**Figure 4:**
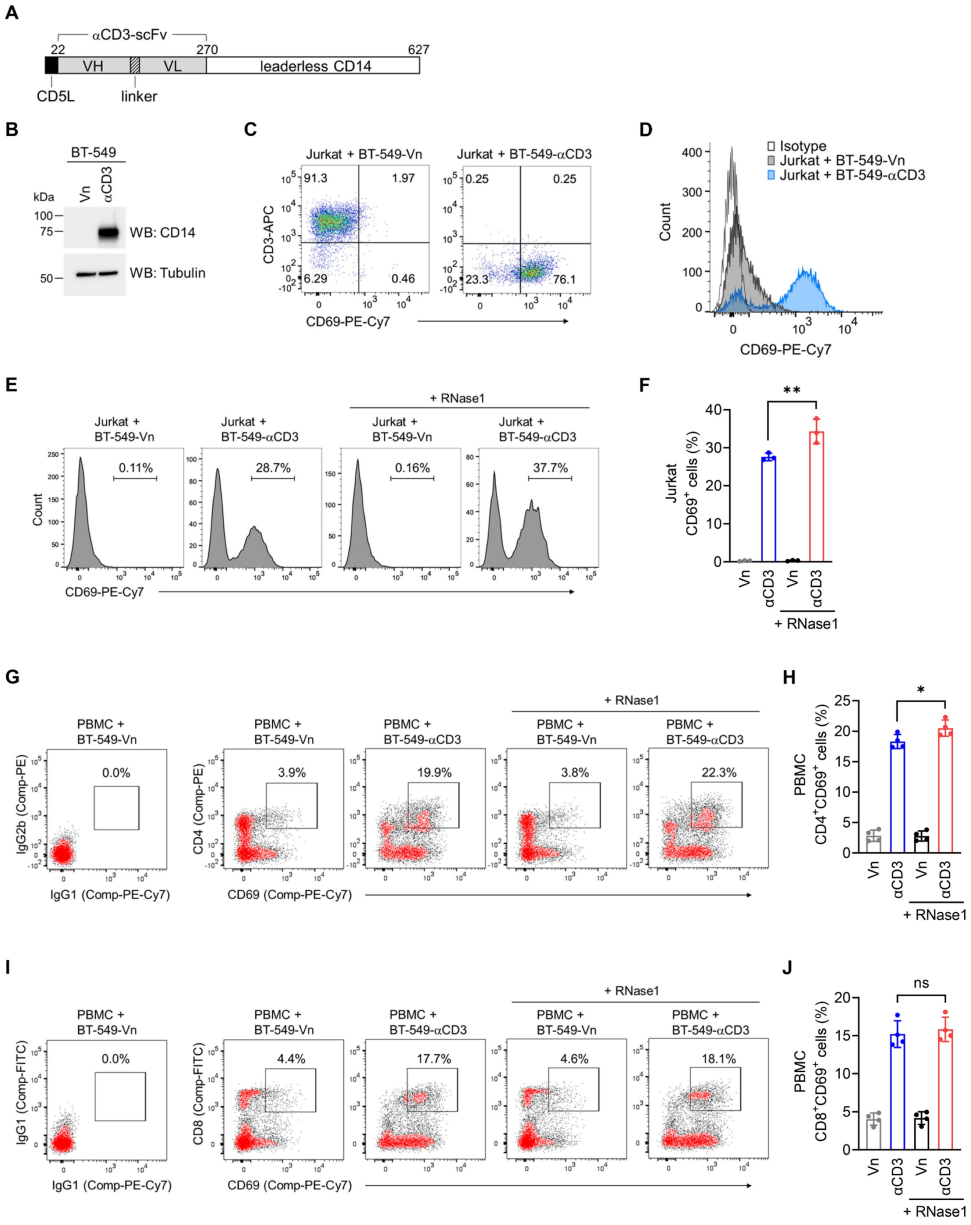
** RNase1 boosts CD4^+^ T cell activation.** (A) Schematic illustration of expression construct encoding anti-CD3 antibody single-chain fragment variable (αCD3-scFv) fused to the transmembrane domain of CD14. The numbers represent amino-acid residues. CD5L, CD5 leader; VH, variable domain of the heavy chain; VL, variable domain of the light chain. (B) Western blotting of the indicated stable cells with antibodies against CD14 and tubulin as loading control. The experiment was repeated a second time with similar results. (C) Flow cytometric analysis of CD69 and CD3 expression in the indicated BT-549 stable clones cocultured with Jurkat cells for 2 days. (D) Flow cytometric analysis of CD69 expression in the indicated BT-549 stable clones cocultured with Jurkat cells for 2 days. IgG isotype control was performed in the BT-549-Vn cocultured with Jurkat cells. (E and F) Representative flow cytometric images (E) and quantitative analysis (F) of CD69 expression in the indicated BT-549 stable clones cocultured with Jurkat cells combined with or without RNase1 treatment (1 μg/ml) for 24 h. (G-J) Representative flow cytometric images and quantitative analysis of the expression of CD69 and CD4 (G and H) or CD69 and CD8 (I and J) in the indicated BT-549 stable clones cocultured with human PBMCs combined with or without RNase1 treatment (1 μg/ml) for 24 h. IgG isotype control, left panel. Data represent the mean ± SD of three independent experiments. *p < 0.05, **p < 0.01, ns, not significant, two-tailed unpaired *t* test.

We hypothesized that RNase1 is enabled to stimulate T cell activation, which triggers T cell-mediated antitumor activity, accounting for the observed antitumor responses. To test this hypothesis, we established a coculture cell-engineered system 31 to interrogate the crosstalk between T cells and tumor cells for the assessment of T cell activation capacity and cancer-killing potential. Briefly, given that T cells are activated via CD3, we generated human breast cancer BT-549 cell lines that stably express an empty vector (BT-549-Vn) or a construct encoding an anti-CD3 antibody fragment fused to the transmembrane domain of CD14 (BT-549-αCD3), and the expression of anti-CD3 was examined with a CD14 antibody (Figure 4A and 4B).

The flow cytometry data indicated that coculture of Jurkat, an immortalized T lymphocyte cell line, with BT-549 tumor cells expressing anti-CD3 resulted in a notable increase of the T cell activation marker CD69, compared to its levels in BT-549-Vn coculture (Figure 4C and 4D), suggesting that T cells were activated via their T cell receptors triggered by anti-CD3 engineered on tumor cells. These results as evidenced by CD69 expression were comparable to those of a widely used approach in which Jurkat cells are stimulated with an CD3/CD28 T cell activator (Figure S4A), further validating our coculture system, allowing for investigation into T cell activation. These engineered tumor cells provide an initial signal to T cells, directly driving T cell activation, which bypasses the need for T cell receptor recognition of an antigenic peptide complexed with major histocompatibility complex on tumor cells 54. The application of a membrane-bound fragment of anti-CD3 antibody was previously used to create immunosomes as artificial virus-like antigen-presenting particles for the stimulation of human T cells 55, and the cell-engineered system has been functionally demonstrated in human lung cancer cells 56.

We employed this established cellular system to determine whether RNase1 takes part in the activation of T cells in the breast TME. As indicated in Figure 4E, coculture of Jurkat with BT-549-αCD3 cells expectedly triggered T cell activation, with a higher percentage of cells expressing CD69 compared to vector control cells (28.7% vs. 0.11%). Strikingly, the addition of recombinant RNase1 protein in the coculture system with BT-549-αCD3 cells further augmented CD69 expression (Figure 4E, 37.7% vs. 28.7%), and a quantification analysis showed a statistically significant enhancement of CD69^+^ cells upon RNase1 treatment (Figure 4F, red vs. blue), suggesting an important role of RNase1 in boosting activated T cells.

To further dissect the effect of RNase1 on CD69 expression in CD4^+^ and CD8^+^ T cell populations, we performed flow cytometric analyses using human peripheral blood mononuclear cells (PBMCs) containing T cells of both CD4 and CD8 lineages, because Jurkat cells lack the expression of endogenous CD8. Indeed, coculture of PBMCs with BT-549-αCD3 compared to that with BT-549-Vn had an increased percentage of CD4^+^CD69^+^ cells (Figure 4G and 4H, blue vs. gray) and CD8^+^CD69^+^ cells (Figure 4I and 4J, blue vs. gray), indicating that the coculture triggered CD4^+^ and CD8^+^ PBMC-T cell activation. However, RNase1 treatment significantly boosted CD69 expression only in the PBMC-T cell subset of CD4^+^ cells (Figure 4G and 4H, red vs. blue), not in the CD8^+^ PBMC-T cells (Figure 4I and 4J, red vs. blue).

These results with the use of recombinant RNase1 protein were further verified in another set of stable clones exogenously expressing RNase1 or the control counterpart of puromycin selection vector in BT-549-Vn (BT-549-Vn-R1 or BT-549-Vn-Vp) or BT-549-αCD3 (BT-549-αCD3-R1 or BT-549-αCD3-Vp) cells (Figure S4B). Consistently, coculture of Jurkat cells with BT-549 cells that coexpressed anti-CD3 fragment and RNase1 had higher levels of CD69^+^ cells than did coculture with BT-549 cells expressing anti-CD3 alone (Figure S4C and S4D, red vs. blue). In line with our previous findings using human PBMCs, we observed an enhancement of CD69 expression in BT-549 cells coexpressing anti-CD3 and RNase1 in the CD4^+^, but not CD8^+^, PMBC-T cells, compared with cells without exogenous RNase1 (Figure S4E to S4G, red vs. blue). These results demonstrated that the presence of RNase1 in the breast TME helps promoting CD4^+^ T cell activation, which may in turn facilitate T cell-mediated eradication of cancer cells.

### RNase1 enhances T cell-mediated killing towards tumor cells

**Figure 5:**
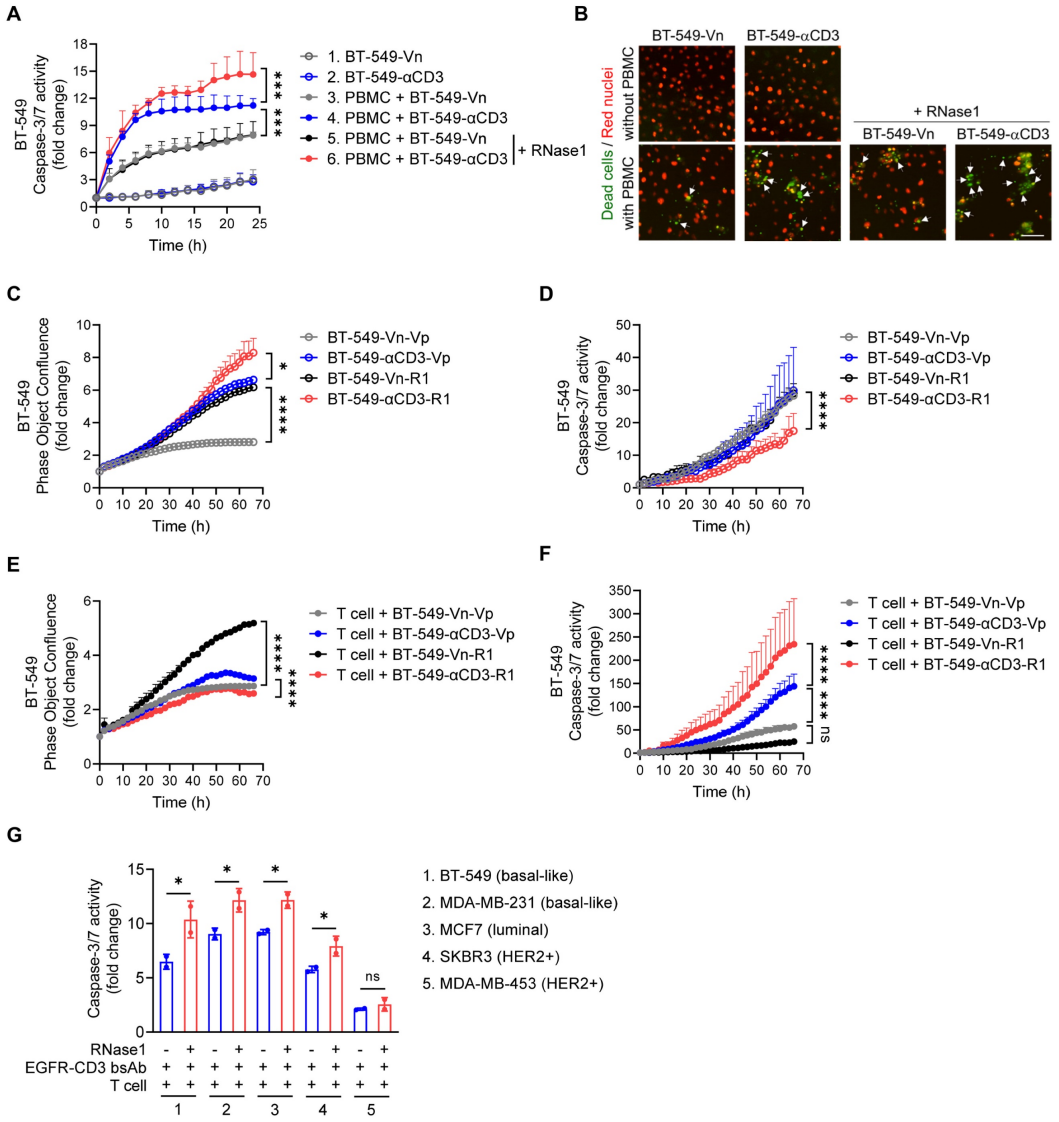
** RNase1 enhances T cell-mediated cell death in breast cancer cells.** (A) Time-course quantitation of T cell-meditated tumor cell-killing assay of dead cells, normalized to that at the zero-time point, in the indicated BT-549 stable cells expressing nuclear-restricted red fluorescent protein cocultured with or without human PBMCs combined with or without RNase1 treatment (1 μg/ml) as indicated. (B) Representative merged images of nuclear-restricted red fluorescent protein (Red nuclei) and green fluorescent caspase 3/7 substrate (Dead cells) from (A) observed at 24 h. Scale bar, 100 μm. Images were captured using the IncuCyte Zoom microscope. (C and D) Time-course quantitation of phase object confluence (C) and dead cells (D), normalized to that at the zero-time point, in the indicated BT-549 stable cells. (E and F) Time-course quantitation of T cell-meditated tumor cell-killing assay of phase object confluence (E) and dead cells (F), normalized to that at the zero-time point, in the indicated BT-549 stable cells cocultured with primary human T cells. (G) Quantitative ratio of T cell-meditated tumor cell-killing assay of dead cells at 70 h in breast cancer cell lines as indicated cocultured with primary human T cells in the presence of EGFR-CD3 bsAb (1 μg/ml) combined with or without RNase1 treatment (1 μg/ml) as indicated. The number of green fluorescent objects were counted and normalized to that at the zero-time point. Data represent mean ± SD (A, C, E, and G) or mean ± SEM (D and F) of two (G) or three (A, C-F) independent experiments. *p < 0.05, ***p < 0.001, ****p < 0.0001, ns, not significant, ANOVA test (A, C-F), one-tailed unpaired *t* test (G).

To test the possibility that RNase1-mediated T cell activation can affect T cell-mediated killing function, as shown in Figure 5A and 5B, we performed a T cell-mediated tumor cell-killing assay by coculturing human PBMCs with BT-549-Vn or BT-549-αCD3 cells expressing nuclear-restricted red fluorescent protein (red nuclei indicated live cells) in the presence of caspase 3/7 substrate treated with or without RNase1 and then measured green fluorescent caspase-3/7 activity, representing apoptotic dead cells.

As expected, BT-549 cell clones without PBMC incubation had minimal caspase-3/7 activity (Figure 5A, lines 1 and 2), and coculture of PBMCs with BT-549-αCD3 had a larger PBMC-T cell-killing effect than did coculture of PMBCs with BT-549-Vn (Figure 5A, line 4 vs. line 3), supporting the idea that activated PBMC-T cells have a role in tumor suppression. Remarkably, treatment with recombinant RNase1 protein in the coculture system between PBMCs and BT-549-αCD3 rendered tumor cells more sensitive to killing by PBMC-T cells (Figure 5A, line 6 vs. line 4), likely owing to more activated T cells boosted by RNase1, whereas there was no significant difference in levels of apoptotic cells in the presence or absence of RNase1 when PBMC-T cells were not activated (Figure 5A, line 5 vs. line 3). The enhanced killing of RNase1 was also observed in cocultures of PBMCs with the above-established BT-549 stable clones exogenously expressing anti-CD3 or RNase1 alone or anti-CD3 combined with RNase1 (Figure S5A).

To further verify the antitumor response played by the activated human PBMC-T cells, we used primary human T cells; consistently, we found that adding RNase1 recombinant protein in the coculture system of primary T and BT-549-αCD3 cells exerted stronger caspase-3/7 activity than did the same system without RNase1 (Figure S5B, red vs. blue). Next, we extensively analyzed the effect of RNase1 on cell proliferation rate in confluence and caspase-3/7 activity in the absence or presence of primary T cells (Figure 5C to 5F). Without T cells' participation, the ectopic expression of RNase1 efficiently increased cancer cell growth in both BT-549-Vn and BT-549-αCD3 stable clones (Figure 5C, black vs. gray, red vs. blue, respectively), and BT-549-αCD3-R1 cells showed the lowest caspase-3/7-mediated cell death compared with the other stable clones (Figure 5D). These results mimic those from an immunocompromised animal model showing a tumor-promoting role in nude mice expressing RNase1 (Figure S1). In contrast, the presence of T cells cocultured with BT-549-αCD3-R1 resulted in a slower proliferation rate and higher caspase-3/7 activity of cancer cells than did the control BT-549-αCD3-Vp without RNase1 expression (Figure 5E and 5F, red vs. blue), suggesting that cells expressing RNase1 gained more sensitivity to killing by activated T cells. Of note, when T cells were cocultured with BT-549-Vn cells, upon which T cell activation was absent, the BT-549-Vn cells expressing RNase1 still showed more cell growth than did the vector control (Figure 5E, black vs. gray). Together, these results strongly support the raised possibility that RNase1-mediated tumor suppression is T cell dependent.

Considering the antitumor role of RNase1 in the breast TME, we then determined whether the therapeutic efficacy of cancer immunotherapies, e.g., T cell-engaging bispecific antibody (T-bsAb) 57, can be improved with the aid of RNase1. To this end, a recombinant T-bsAb against EGFR on tumor cells and CD3 on T cells (EGFR-CD3 bsAb) was used to perform T cell-mediated killing in a coculture system of primary T cells with a panel of breast cancer cell lines, including basal-like (BT-549 and MDA-MB-231), luminal (MCF7), and HER2^+^ (SKBR3 and MDA-MB-453) subtypes, in the presence or absence of RNase1 (Figure 5G). Strikingly, the addition of RNase1 enhanced the EGFR-CD3 bsAb-triggered caspase-3/7 activity in all the cells except in an EGFR-negative MDA-MB-453 cell line (Figure 5G). This finding suggests that RNase1 and EGFR-CD3 bsAb collaborate to protect against breast cancer across different molecular subtypes 58, which may provide a rational strategy for immunocompetent patients with breast cancer by combining RNase1 with T-bsAb against specific antigens.

### EphA4 on T cells may contribute to RNase1-mediated antitumor immunity

**Figure 6:**
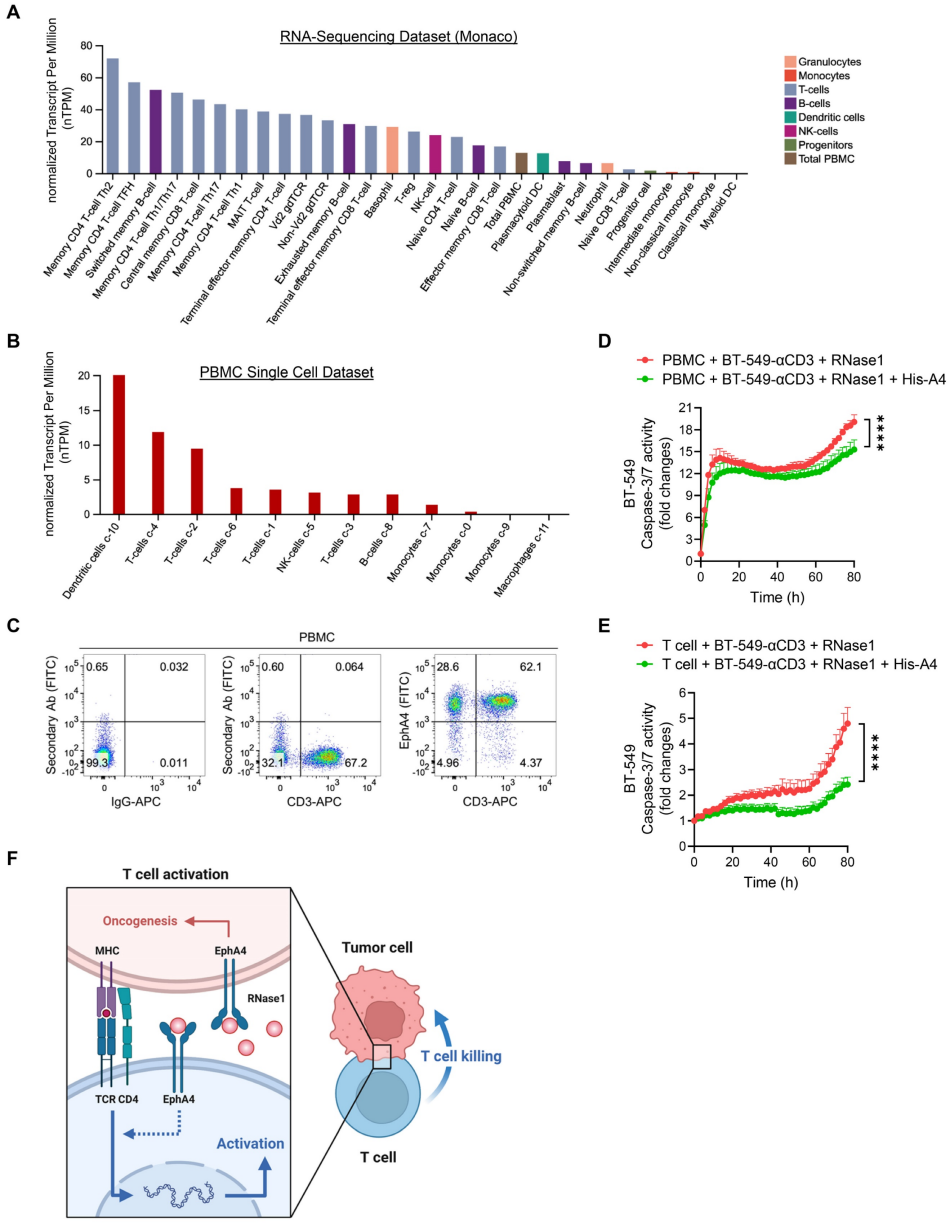
** EphA4 on T cells may contribute to RNase1**-**mediated antitumor immunity.** (A) Quantitative analysis of RNA-sequencing results from Human Protein Atlas (Monaco database) of EphA4 expression on immune cells. (B) Quantitative analysis of EphA4 gene expression on a single-cell level of human PBMCs based on markers of different cell type lineages. (C) Flow cytometric analysis of EphA4 and CD3 expression in human PBMCs. IgG-APC and secondary Ab only serve as negative controls. Frequencies of cell population after gating are indicated in the quadrant of each panel. (D and E) Time-course quantitation of T cell-meditated tumor cell-killing assay of dead cells, normalized to that at the zero-time point, in BT-549-αCD3 cells cocultured with human PBMCs (D) or primary T cells (E) combined with RNase1 treatment (1 μg/ml) in the presence or absence of recombinant His-A4 (5 μg/ml) as indicated. Data represent mean ± SEM of three independent experiments. ****p < 0.0001, ANOVA test. (F) A proposed model of RNase1-mediated T cell killing towards tumor cells in the breast TME. In brief, serum RNase1 known as a ligand of EphA4 promotes oncogenesis in breast cancer cells. However, T cell function plays a dominate role in the presence of the immune system, namely that RNase1 boosts CD4^+^ T cell activation, in which EphA4 may participate, leading to activated T cell-mediated antitumor immunity. Artwork, adapted from “T-cell Deactivation vs. Activation”, by BioRender.com (2023). Retrieved from https://app.biorender.com/biorender-templates

As EphA4 is a cognate receptor of RNase1 via a direct ligand-receptor binding 24, we next determined, in addition to its expression on tumor cells, whether EphA4 was also expressed on immune cells in the TME, where it may interact with secretory RNase1 and be involved in RNase1-mediated antitumor function. Previous studies have reported EphA4 expression on T cell populations at the mRNA and protein levels 29, 59. Indeed, on the basis of RNA-sequencing results from two independent datasets (Human Protein Atlas) 34, 35, EphA4 was expressed on a variety of immune cells, with CD4 T cell subsets among the top ranked (Figure 6A and S6A). Furthermore, we launched into EphA4 expression in the Human Protein Atlas database of PBMCs analyzed on a single-cell level to have a heatmap based on well-known markers of cell type lineages along with their quantifications, which showed the relatively high expression of EphA4 in T cell populations (Figure 6B and S6B). Similar patterns of high EphA4 expression on T cells were observed in another dataset of PBMCs separated into subpopulations by flow sorting (Figure S6C). Together with our flow cytometry data indicating an enriched EphA4^+^CD3^+^ T cell population in PBMCs (Figure 6C and S6D), the existence of the RNase1-EphA4 axis on T cells may play a part in upregulating T cell-mediated antitumor activity, which is distinct from the dominant role of this axis in tumor cells as an oncogenic pathway 24. Interestingly, pre-incubation of the coculture system between PBMCs or primary T cells and BT-549-αCD3 tumor cells with the recombinant extracellular domain of EphA4 (His-A4), which can neutralize EphA4 ligands such as RNase1, significantly decreased RNase1-mediated killing ability towards tumor cells (Figure 6D and 6E). Taken together, these results suggest that RNase1 bound to its cognate receptor EphA4 on T cells at least partially participates in T cell-mediated antitumor immunity. A future study is warranted to investigate the unique function and underlying mechanism of the RNase1-EphA4 axis on immune cells, in particular T cells, in the TME.

## Discussion

Based on our findings, we propose a model of the breast TME in which RNase1 freely circulating in the blood acts as an alarmin to elicit adaptive immune response by boosting CD4^+^ T cell activation, resulting in activated T cell-mediated antitumor immunity (Figure 6F). Administration of RNase1 in the presence of the immune system may effectively modulate both aspects of the innate and adaptive immune systems in cooperation. Considering the contribution of the immune system to tumor development and progression, accumulating evidence indicates that results obtained from different mouse models, namely models of immunodeficiency and immunocompetence, may be quite different 60. For example, MET inhibitors suppress liver tumor development in nude mice yet lose tumor suppressive activity in immunocompetent mice due to upregulation of PD-L1 expression 61. In addition, lymphocyte-deficient mice are more susceptible than immunocompetent mice to sarcoma development induced by the chemical carcinogen methylcholanthrene 62. Moreover, inhibition of indoleamine 2,3-dioxygenase-1, an enzyme that catalyzes the degradation of an essential amino acid tryptophan, suppresses tumor growth in a lymphocyte-dependent manner in immunocompetent but not immunocompromised mice bearing pancreatic tumors 63.

Our results in immunocompromised nude mice bearing 4T1 breast tumors showed that RNase1 promoted tumor weight 24 and tumor volume (Figure S1) by associating with EphA4, which is distinct from the results of Fischer et al., who found that RNase1 inhibits tumor growth in nude mice bearing HT-29 colon tumors through the reduction of exRNAs-mobilized tumor necrosis factor-α 23. The discrepancy may mainly come from tumor or cell-type specificity because HT-29 cells, unlike 4T1 cells, express low or undetectable levels of EphA4 64, thus stopping RNase1 from binding to and activating EphA4 signaling in HT-29 cells. High expression levels of EphA4 have been observed in various cancers associated with oncogenic functions 65. As we know, a number of cancer patients have a weakened immune system and are considered immunocompromised, which could mimic the conditions of our findings in nude mice demonstrating a tumor-promoting role of RNase1 through EphA4 ligand-receptor interaction. In contrast with the oncogenic role of EphA4 that is well-documented on tumor cells, the role of EphA4 on immune cells remains ill-defined. EphA4 expression is reported in subpopulations of CD4^+^ T, CD8^+^ T, and CD19^+^ B cells by flow cytometry analysis, although another report mentioned that EphA4 mRNA is undetectable on CD8^+^ T cells by Northern blot analysis 66. EphA4 after binding to ephrin-A1 ligand facilitates the migration of CD4^+^ T cells, in particular the CD4^+^CD45RO^+^ memory subset of T cells. It would be worthwhile to elucidate whether the binding of RNase1 to EphA4 on CD4^+^ T cells stimulates their migration to tumor tissue for maintaining immune homeostasis.

In our studies, RNase1 boosted T cell activation preferentially on CD4^+^ T cells (Figure 4 and S4). Notably, pre-clinical and clinical studies have clarified the roles of cytotoxic CD4^+^ T cells in various human cancer types, including breast cancer, in which intra-tumoral CD4^+^ T cells that express cytolytic proteins, such as granzymes, can directly kill tumor cells independently of conventional CD8^+^ T cells 67. Moreover, studies of the interplay between CD4^+^ T and NK cells and how those interactions regulate antitumor immunity have also gradually emerged 46, 53. As proof, newly primed antigen-specific CD4^+^ T cells were shown to initiate NK cell activation through the secretion of interleukin (IL)-2 68. Additionally, IL-17, a proinflammatory cytokine primarily produced by CD4^+^ Th17 cells, exerts an antitumor effect by recruiting CD4^+^ T, CD8^+^ T, and NK cells to the tumor site, enhancing NK cell activity 47. Given that RNase1 increases cell frequencies of CD4^+^ Th1, Th17, and NK cells (Figure 2 and 3), it is possible that RNase1 increases CD4^+^ T cell-specific subsets, which in turn activate NK cell function to generate a more robust immune response for eradicating tumor cells. Future investigations into such a possibility of NK cell participation are required to reveal more detailed mechanistic insights about RNase1 in antitumor immunity.

Raines and colleagues have developed an engineered variant of the RNase1 protein that targets RNAs in cancer cells by diminishing the protein's association with a natural RNase inhibitor in the cytosol, which is undergoing clinical trials as an anti-cancer agent in non-small cell lung and ovarian cancers 69. Other than the RNase1-mediated cytotoxic effect through RNA degradation, our findings provide information about an additional mechanism by which RNase1 enhances T cell activation in regulating breast tumor shrinkage, likely through the association with EphA4 on T cells in the TME, in which the ligand-receptor interaction is known to be independent of the RNase's ribonucleolytic activity 24, 70. Nowadays, cancer immunotherapy using bispecific antibodies (bsAb) has demonstrated potent efficacy and has been approved for the treatment of hematologic malignancies while numerous bsAb for solid tumors are at various stages of clinical trials 71-73. Notably, the effectiveness of EGFR-CD3 bsAb has been shown in preclinical studies, including those on glioblastoma, pancreatic, and colon cancers 74-77; for these tumor models, our findings (Figure 5G) suggest a synergistic antitumor effect when bsAb are combined with RNase1 treatment. Interestingly, we detected increased levels of PD-1^+^ T cells in response to RNase1 (Figure 3E), suggesting that a combination therapy of RNase1 with anti-PD-1/PD-L1 immunotherapy may be worthwhile or that RNase1 status serves as a non-invasive biomarker to stratify patients, with those who have high serum levels of RNase1 possibly benefitting from anti-PD-1/PD-L1 immunotherapy optionally combined with EphA4 inhibition.

In summary, our findings provide insights into the functional role of RNase1 in the adaptive immune system by enhancing T cell activation to eliminate tumor growth. Augmenting antitumor immunity by RNase1 addition represents a promising therapeutic strategy against breast cancer. Given that EphA4 serves as a cognate receptor of RNase1 in breast cancer, furthering our understanding of EphA4 on T cells is worth pursuing to expand the development of cancer treatments by targeting the RNase1-EphA4 axis in the immune system.

## Supplementary Material

Supplementary figures.

## Abbreviations

RNase: ribonuclease
TME: tumor microenvironment
HER2: human epidermal growth factor receptor 2
RTK: receptor tyrosine kinase
EphA4: ephrin receptor A4
EGFR: epidermal growth factor receptor
CyTOF: cytometry by time-of-flight
NK cells: natural killer cells
MDSCs: myeloid-derived suppressor cells
DCs: dendritic cells
Th cells: T helper cells
Treg cells: regulatory T cells
PD-1: programmed cell death 1
LAG-3: lymphocyte activation gene 3
TIM-3: T cell immunoglobulin mucin-3
PBMCs: peripheral blood mononuclear cells
