# Supplementary material for: Ribonuclease 1 Enhances Antitumor Immunity against Breast Cancer by Boosting T cell Activation
Source: Int J Biol Sci. 2023 Jun 4;19(10):2957–73. doi: 10.7150/ijbs.84592 (PMC10321278; doi:10.7150/ijbs.84592)
Supplement: Supplementary file 1 — Supplementary figures. [file ijbsv19p2957s1.pdf]

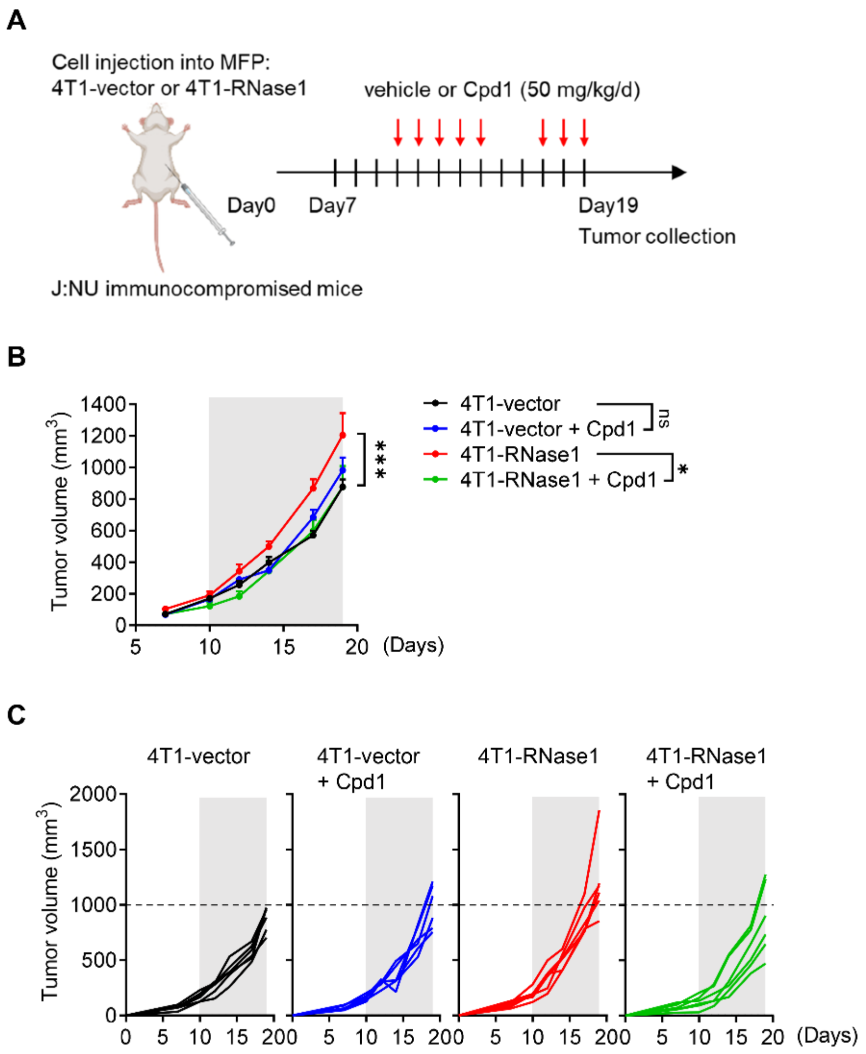

Figure S1. RNase1 ectopic expression enhances tumor progression in an immunocompromised nude mouse model. (A) The schedule of treatment in J:NU mice orthotopically injected with the indicated 4T1 cells into the mammary fat pad (MFP) followed by treatment with vehicle or compound 1 (Cpd1). The arrows indicate the time of treatment. n = 6 mice per group. (B and C) Average tumor volume (B) and tumor growth curve (C) of 4T1 cells in J:NU mice treated with vehicle or Cpd1 as indicated. Tumor volume was measured at the indicated time points and dissected at the end point. The gray box indicates the duration of treatment. Data represent mean ± SEM. *$p < 0.05$, ***$p < 0.001$, ns, not significant, ANOVA test.

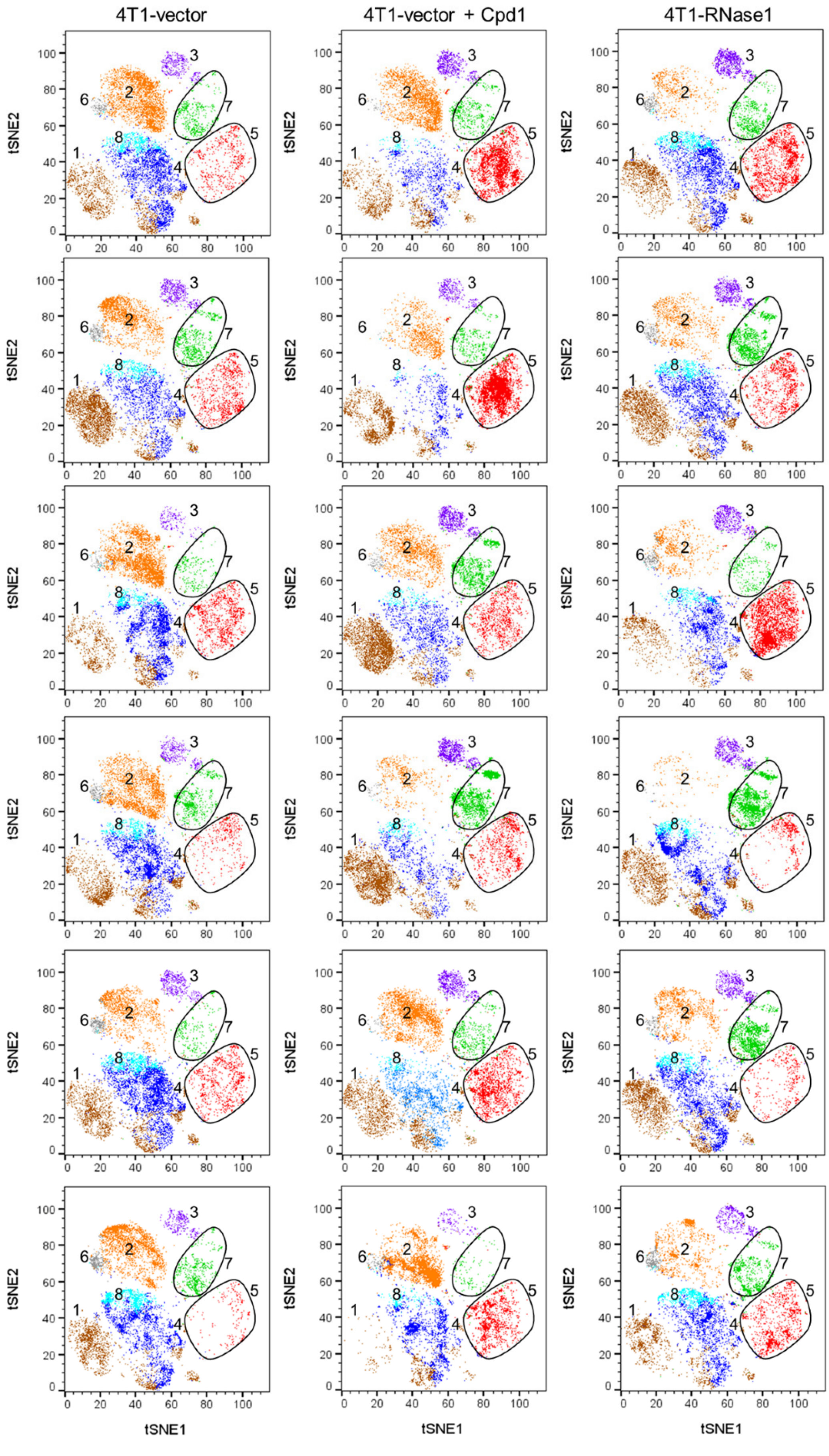
4T1-vector
4T1-vector + Cpd1
4T1-RNase1
1
2
3
4
5
6
7
8
tSNE1
tSNE2
0
20
40
60
80
100

Figure S2. CyTOF analysis of tumor-infiltrating immune cells of mice with the established 4T1 tumors. t-SNE and FlowSOM analyses generated eight color-coded clusters for tumor-infiltrating immune cells isolated from each sample (n = 6 in each group). Ten thousand cells are displayed in each t-SNE plot.

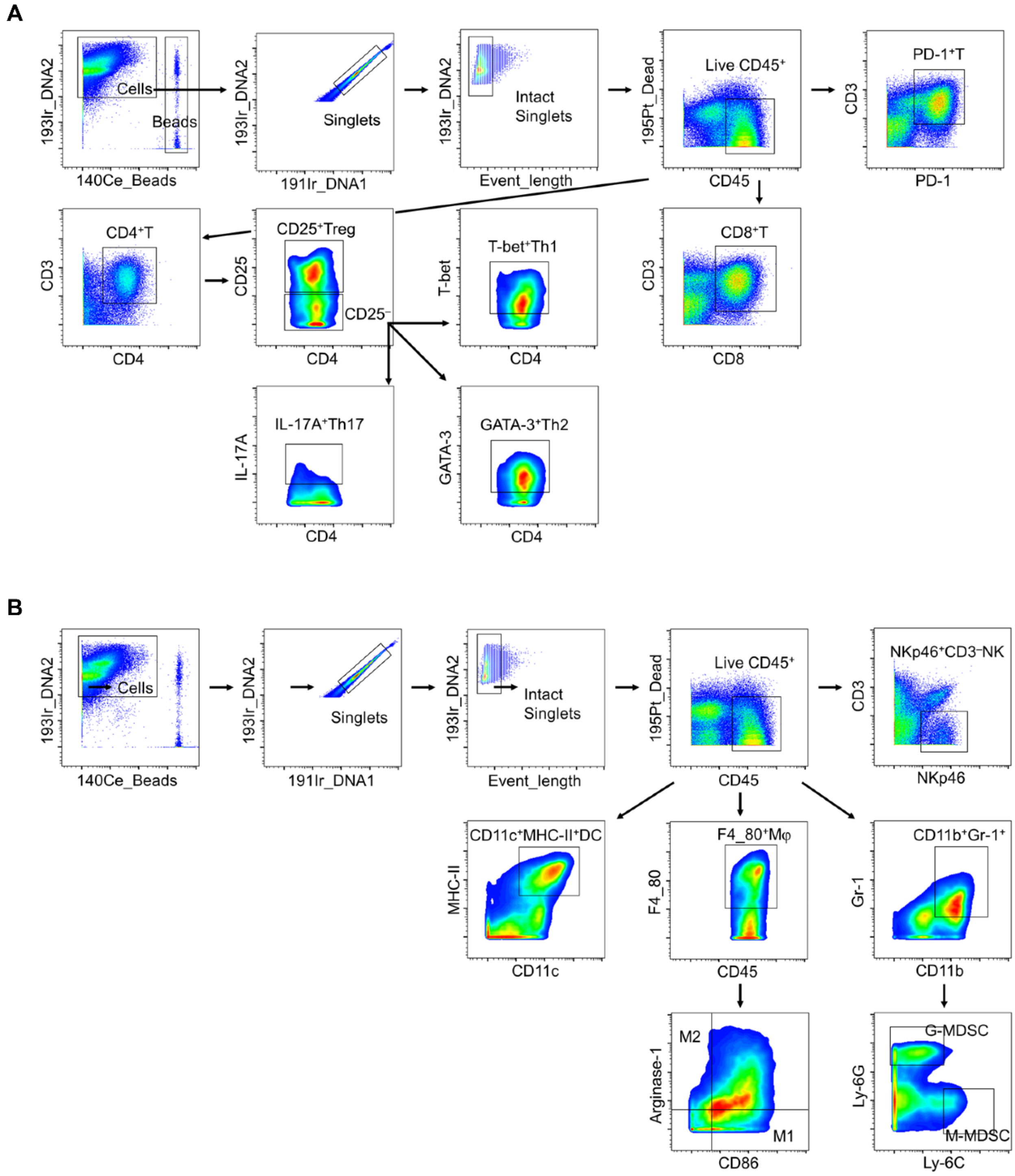

Figure S3. A conventional hand-gated strategy used for CyTOF analysis. (A and B) Gating strategies to determine the percentage of the indicated cell populations in mice tumors presented on Figure 3A–3E (A) and Figure 3F–3I (B).

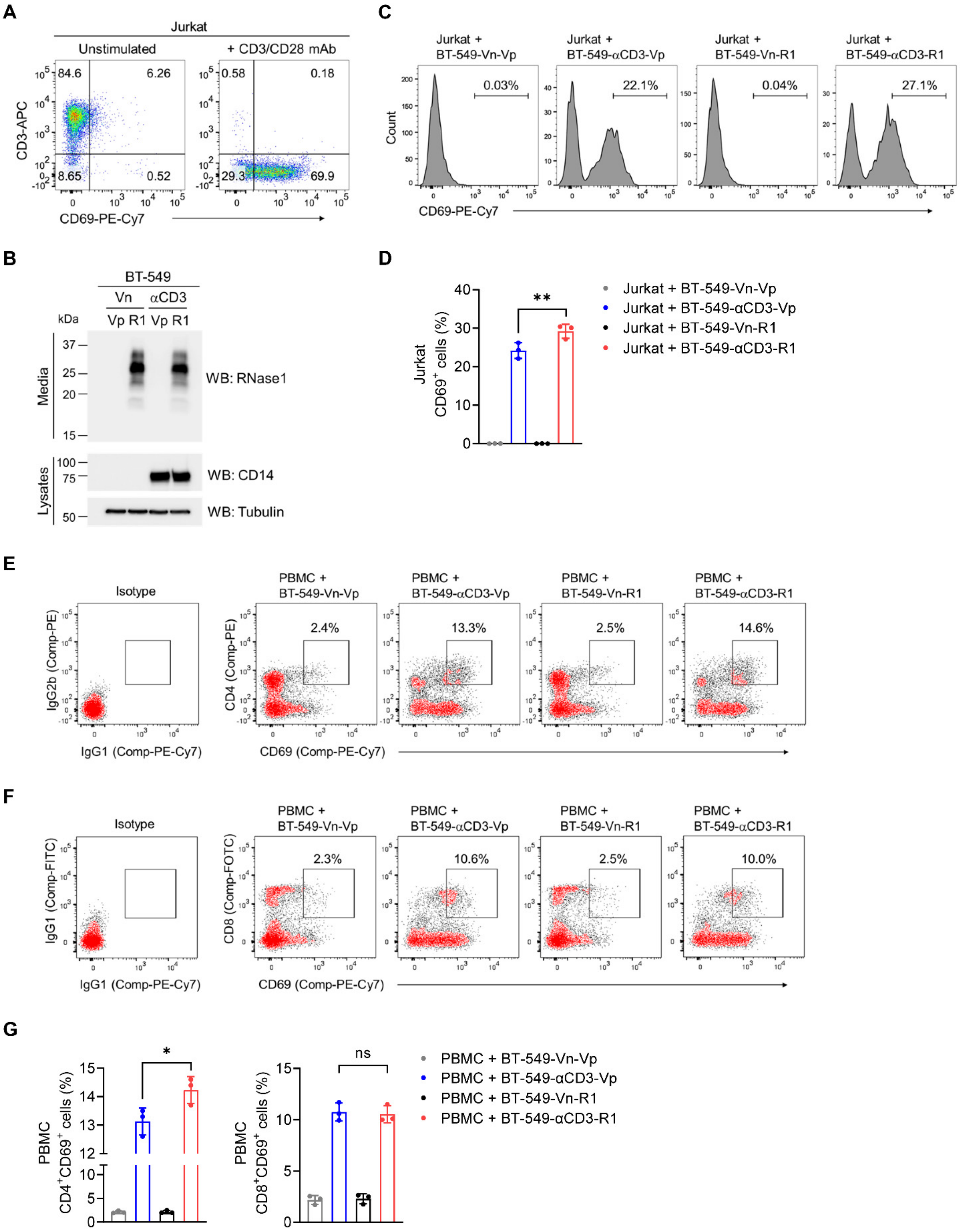
A
Jurkat
Unstimulated
+ CD3/CD28 mAb
CD3-APC
CD69-PE-Cy7
84.6
6.26
8.65
0.52
0.58
0.18
29.3
69.9
B
BT-549
Vn
αCD3
Vp R1 Vp R1
kDa
Media
Lysates
WB: RNase1
WB: CD14
WB: Tubulin
C
Jurkat + BT-549-Vn-Vp
Jurkat + BT-549-αCD3-Vp
Jurkat + BT-549-Vn-R1
Jurkat + BT-549-αCD3-R1
Count
CD69-PE-Cy7
0.03%
22.1%
0.04%
27.1%
D
Jurkat CD69+ cells (%)
**
Jurkat + BT-549-Vn-Vp
Jurkat + BT-549-αCD3-Vp
Jurkat + BT-549-Vn-R1
Jurkat + BT-549-αCD3-R1
E
Isotype
IgG2b (Comp-PE)
IgG1 (Comp-PE-Cy7)
PBMC + BT-549-Vn-Vp
PBMC + BT-549-αCD3-Vp
PBMC + BT-549-Vn-R1
PBMC + BT-549-αCD3-R1
CD4 (Comp-PE)
CD69 (Comp-PE-Cy7)
2.4%
13.3%
2.5%
14.6%
F
Isotype
IgG1 (Comp-FITC)
IgG1 (Comp-PE-Cy7)
CD8 (Comp-FOTC)
CD69 (Comp-PE-Cy7)
2.3%
10.6%
2.5%
10.0%
G
PBMC CD4+CD69+ cells (%)
PBMC CD8+CD69+ cells (%)
*
ns
PBMC + BT-549-Vn-Vp
PBMC + BT-549-αCD3-Vp
PBMC + BT-549-Vn-R1
PBMC + BT-549-αCD3-R1

Figure S4. Exogenous expression of RNase1 enhances CD4$^+$ T cell activation. (A) Flow cytometric analysis of CD69 and CD3 expression in Jurkat cells stimulated with or without ImmunoCult human CD3/CD28 T cell activator (STEMCELL Technologies, #10971) for 3 days. (B) Western blotting of secreted proteins from conditioned media (top) and cell lysates (bottom) in the indicated stable cells with antibodies against RNase1, CD14, and tubulin. The experiment was repeated a second time with similar results. (C and D) Representative flow cytometric images (C) and quantitative analysis (D) of CD69 expression in the indicated BT-549 stable clones cocultured with Jurkat cells for 24 h. (E and F) Representative flow cytometric images of CD69 and CD4 (E) or CD69 and CD8 (F) expression in the indicated BT-549 stable clones cocultured with Jurkat cells for 24 h. IgG isotype control, left panel. (G) Quantitative analysis of CD69 and CD4 (left) or CD69 and CD8 (right) expression from (E) and (F), respectively. Data represent the mean ± SD of three independent experiments. *$p < 0.05$, **$p < 0.01$, ns, not significant, two-tailed unpaired *t* test.

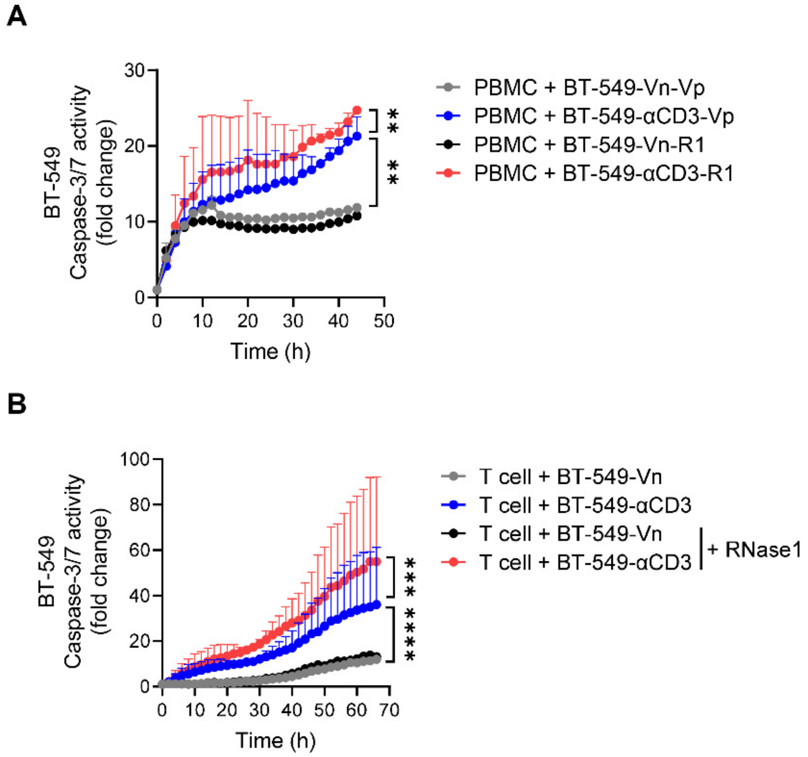

Figure S5. RNase1 enhances T cell-mediated killing towards BT-549 tumor cells. (A) Time-course quantitation of T cell-meditated tumor cell-killing assay of dead cells, normalized to that at the zero-time point, in the indicated BT-549 stable cells cocultured with human PBMCs. (B) Time-course quantitation of T cell-meditated tumor cell-killing assay of dead cells, normalized to that at the zero-time point, in the indicated BT-549 stable cells expressing nuclear-restricted red fluorescent protein cocultured with primary human T cells combined with or without RNase1 treatment (1 μg/ml). Data represent mean ± SEM of three independent experiments. **p < 0.01, ***p < 0.001, ****p < 0.0001, ANOVA test.

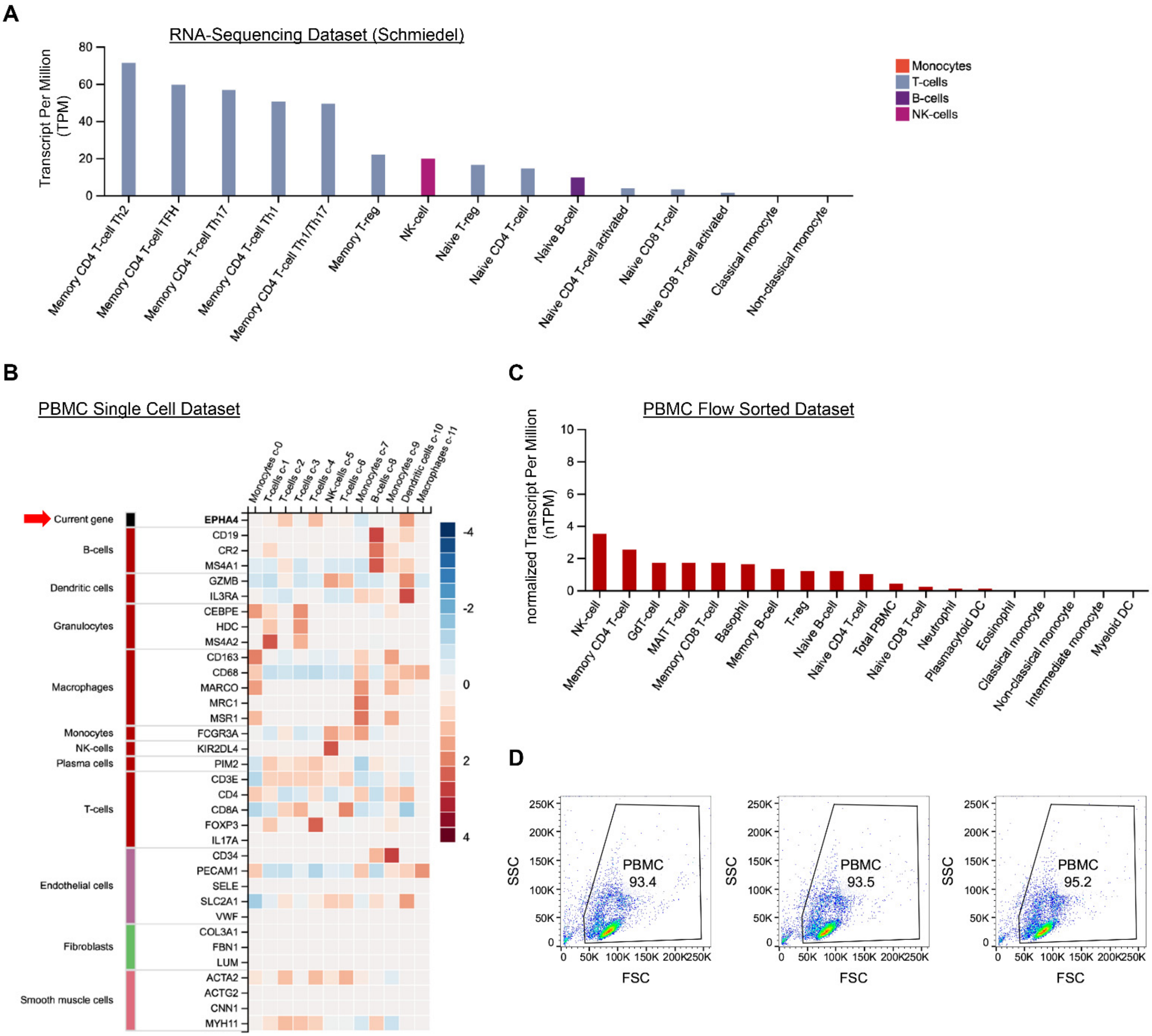

Figure S6. Analysis of EphA4 expression from databases. (A) Quantitative analysis of RNA-sequencing results from Human Protein Atlas (Schmiedel database) of EphA4 expression on immune cells. (B) A heatmap of EphA4 gene expression on a single-cell level of human PBMCs based on markers of different cell type lineages, related to Figure 6B. (C) Quantitative analysis of EphA4 gene expression of human PBMCs separated into subpopulations by flow sorting. (D) Gating strategies based on FSC/SSC parameters used for flow cytometric analysis to determine

the percentage of CD3 and EphA4 expression in PBMC presented on Figure 6C. Frequencies of cell population after gating are indicated in the gates.
